# Molecular Basis of VCPIP1 and P97/VCP Interaction Reveals Its Functions in Post‐Mitotic Golgi Reassembly

**DOI:** 10.1002/advs.202403417

**Published:** 2024-09-05

**Authors:** Tianzhui Liao, Ruotong Li, Ping Lu, Yusong Liu, Rong Yang, Hao Guo, Zhuoxi Wu, Ruiwen Wang, Ling Yuan, Zhengmao Hu, Haishan Gao, Faxiang Li

**Affiliations:** ^1^ MOE Key Laboratory of Rare Pediatric Diseases Center for Medical Genetics School of Life Sciences Central South University Changsha Hunan 410013 China; ^2^ Zhejiang Key Laboratory of Structural Biology Westlake University Hangzhou Zhejiang 310024 China; ^3^ Westlake Laboratory of Life Sciences and Biomedicine Hangzhou Zhejiang 310024 China; ^4^ State Key Laboratory of Developmental Biology of Freshwater Fish Engineering Research Center of Polyploid Fish Reproduction and Breeding of the State Education Ministry College of Life Sciences Hunan Normal University Changsha Hunan 410013 China

**Keywords:** golgi apparatus, membrane fusion, P97/VCP ATPase, SNARE complex, VCPIP1

## Abstract

The VCPIP1‐P97/VCP (Valosin‐Containing Protein) complex is required for post‐mitotic Golgi cisternae reassembly and maintenance in interphase. However, the organization and mechanism of this complex in regulating Golgi membrane fusion is still elusive. Here, the cryo‐electron microscopy (cryo‐EM) structures of the human VCPIP1‐P97/VCP complex are presented. These studies reveal that three independent VCPIP1 molecules sit over the C‐terminal substrate exit tunnel formed by P97/VCP homo‐hexamer, resulting in an unusual C3 to C6 symmetric barrel architecture. The UFD1 (unknown function domain 1) from VCPIP1, but not the N‐terminal OTU domain and the C‐terminal UBL domain, docks to the two adjacent D2 domains of P97/VCP, allosterically causing the cofactors binding domain‐NTDs (N‐terminal domains) of P97/VCP in a “UP” and D1 domain in an ATPase competent conformation. Conversely, VCPIP1 bound P97/VCP hexamer favors the binding of P47, and thus the intact SNARE complex, promoting Golgi membrane fusion. These studies not only reveal the unexpected organization of humanVCPIP1‐P97/VCP complex, but also provide new insights into the mechanism of VCPIP1‐P97/VCP mediated Golgi apparatus reassembly, which is a fundamental cellular event for protein and lipid processing.

## Graphical abstract

This study investigates the molecular organization and mechanism of the VCPIP1‐P97/VCP complex in regulating post‐mitotic Golgi membrane fusion. The cryo‐EM structure of the VCPIP1‐P97/VCP complex reveals an unusual C3 to C6 symmetric barrel architecture, where the UFD1 domain of VCPIP1 docks to P97/VCP, allosterically modulating its conformation to favor the binding of P47 and the SNARE complex, thereby promoting Golgi reassembly.

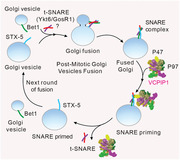

## 1 Introduction

The Golgi apparatus, one of the major cellular membranous organelles composed of unique and complex interconnected tubular and cisternal structures, plays essential processing roles in protein and lipid modification, trafficking, and sorting in eukaryotes.^[^ ^1^, ^2^ ^]^ The structural changes and functional disorders of the Golgi apparatus are involved in many human diseases such as cardiovascular diseases, ischemic stroke, infectious diseases, neurodegenerative diseases, and cancer.^[^ ^3^, ^4^ ^]^ The highly dynamic Golgi apparatus undergoes substantial morphological remodeling in the cell cycle. During the onset of mitosis, the Golgi apparatus disassembles from ribbon‐like stack structures known as Golgi cisternae fragmented into tubulovesicles, and then divide equally into the daughter cells.^[^ ^5^ ^]^ Subsequently, the tubulovesicles fuse and reform the intact and functional stacks congregating in the pericentriolar region during the late telophase.^[^ ^6^ ^]^

Similar to the other cellular membrane vesicles, the fusion of Golgi tubulovesicles is also regulated by the SNARE complex. The syntaxin‐5 (t‐SNARE) and Bet1 (v‐SNARE) composed SNARE complex is responsible for the fusion of fragmented Golgi vesicles in the late stage of mitosis.^[^ ^7^, ^8^ ^]^ The in vitro Golgi reassembly assay revealed that at least two AAA+ ATPases, NSF (N‐ethylmaleimide–Sensitive factor), and P97/VCP, play crucial roles in the Golgi fusion process from fragmented tubulovesicles.^[^ ^9^, ^10^ ^]^ However, there are some differences in the functions of NSF and P97/VCP in cellular membrane fusion.^[^ ^11^ ^]^ NSF is mainly responsible for the fusion between membrane vesicles from different sources, such as the membrane fusion between the endoplasmic reticulum and the Golgi apparatus. P97/VCP is mainly responsible for the fusion between membrane vesicles of the same source, such as the membrane vesicle fusion within the Golgi apparatus.^[^ ^12^ ^]^ The molecular mechanism by which NSF regulates membrane fusion has been well characterized. NSF binds to SNARE complexes via adapter protein α‐SNAP, and then dissociates the SNARE complex through ATP hydrolysis, thereby separating the v‐ and t‐SNAREs.^[^ ^13^ ^]^ This process is called SNARE priming. Once the SNAREs are primed, they can readily engage with SNAREs from other vesicles, facilitating further rounds of fusion. Due to the similar structure and unfoldase activity with NSF, P97/VCP is also thought to prime the SNAREs via ATP hydrolysis.^[^ ^14^, ^15^ ^]^ But until now, the molecular mechanism of P97/VCP in regulating Golgi membrane fusion remains elusive.

P97/VCP is an ATP‐dependent unfoldase that forms a homo‐hexamer machinery extracting ubiquitinated or non‐ubiquitinated proteins from macromolecular complexes, membranes, and misfolded protein aggregates for subsequent degradation or remodeling.^[^ ^16^, ^17^ ^]^ In complex with various adaptors, P97/VCP regulates diverse cellular physiological processes, including endoplasmic reticulum‐associated degradation (ERAD) membrane fusion, and DNA replication and repair.^[^ ^18^, ^19^ ^]^ The cofactors P47 and P37 form tight complexes with P97/VCP, which are essential for ER and Golgi biogenesis. The P97/VCP‐P37 pathway‐mediated ER and Golgi biogenesis mainly occurs in interphase cells and utilizes the p115‐GM130 tethering complex.^[^ ^14^ ^]^ On the contrary, the P97/VCP‐P47 pathway specifically regulating the organelle's reassembly mostly occurs at the end of mitosis.^[^ ^20^ ^]^ A recent study uncovered that P97/VCP/P47 binds to the FTCD (formiminotransferase cyclodeaminase) to form the P97/VCP‐FTCD/P47‐FTCD tethering complex during the Golgi reassembly process at the end of mitosis.^[^ ^21^ ^]^ Different from the NSF‐α‐SNAP pathway, P97/VCP‐P47 is unable to dissociate the SNARE complex in vitro, even in the presence of ATP. VCPIP1(VCP135, Valosin‐Containing Protein‐Interacting Protein, p135), a P97/VCP‐P47 complex interacting protein, is strictly required for both ER and Golgi membrane fusion processes regulated by P97/VCP‐P47.^[^ ^15^ ^]^

VCPIP1, first identified as P97/VCP interacting protein, is a multi‐domain containing deubiquitinase (DUB), which harbors an N‐terminal OTU domain, a middle UFD1 domain, and the C‐terminal UBL domain. Knock down the expression of the VCPIP1 gene by siRNA resulted in the Golgi fragmentation, resembling the phenotype by injection of the specific P97/VCP or P47 antibodies into cells to block the function of the P97/VCP‐P47 pathway.^[^ ^22^, ^23^ ^]^ The cytosolic protein VCPIP1 directly binds to the P97/VCP‐P47‐SNAREs complex and dissociates it through P97/VCP‐mediated ATP hydrolysis, thereby facilitating the Golgi vesicle fusion in late mitosis.^[^ ^15^ ^]^ The deubiquitinase activity of VCPIP1 was reported to be essential for the P97/VCP‐P47 mediated Golgi membrane fusion, but not for the P97/VCP‐P37 pathway.^[^ ^24^ ^]^ The deubiquitination activity of VCPIP1 was precisely controlled by a phosphorylation switch coupled to the cell cycle. VCPIP1 S130 was phosphorylated by Cdk1 in early mitosis and then inactivated to block the P97/VCP‐P47 mediated Golgi membrane fusion. This site was dephosphorylated in late mitosis and VCPIP1 was activated to promote the Golgi reassembly.^[^ ^25^, ^26^, ^27^ ^]^ The function of t‐SNARE syntaxin‐5 in Golgi membrane fusion was precisely regulated by the ubiquitination process. The K270 on the SNARE domain of syntaxin‐5 was mono‐ubiquitinated by the ubiquitin ligase HACE1 in early mitosis and deubiquitinated by VCIPIP1 at the end of mitosis. The ubiquitination of syntaxin‐5 decreased its interaction with the cognate v‐SNARE Bet1, thereby impairing the Golgi membrane fusion.^[^ ^7^ ^]^ In all, VCPIP1 regulates the post‐mitotic Golgi reassembly through ubiquitin‐dependent and independent pathways. A very recent study reported that the N‐ and C‐terminal of VCPIP1 are both required for the P97/VCP binding, and the two binding sites on VCPIP1 are essential for the post‐mitotic Golgi membrane fusion.^[^ ^28^ ^]^ However, the molecular mechanism of VCPIP1 interacting with P97/VCP and the precise roles of the VCPIP1‐P97/VCP complex in Golgi membrane fusion in late mitosis is still elusive.

In this study, we first analyzed the interactions between P97/VCP and VCPIP1 fragments and found out that VCPIP1 can associate with P97/VCP in vitro through multiple binding sites, including the N‐terminal OTU domain, the middle UFD1 domain, and the C‐terminal UBL‐UFD2 domain. Moreover, we successfully determined the Cryo‐EM structure of the full‐length P97/VCP‐VCPIP1 complex, which reveals that three individual VCPIP1 molecules directly bind to the C‐terminal D2 domains of the P97/VCP hexamer through their UFD1 domains. The VCPIP1 binding promotes the P97/VCP into the NTDs “UP” conformation suggesting the regulation of the unfoldase activity of P97/VCP by VCPIP1. The biochemical data demonstrated that VCPIP1 binding significantly enhanced the affinity of P97/VCP for its SNARE complex substrates. Additionally, the cell‐based immunofluorescence assay confirmed that the interaction between VCPIP1 UFD1 and P97/VCP D2 is essential for the Golgi fragmented tubulovesicles fusion during the cell cycle. Overall, our study not only reveals the unexpected interaction and organization of human P97/VCP‐VCPIP1 complex, but also provides crucial insights into the mechanism of VCPIP1‐P97/VCP mediated Golgi biogenesis.

## 2 Results

### 2.1 VCPIP1 Associates with P97/VCP through Multiple Binding Sites

**Figure 1:**
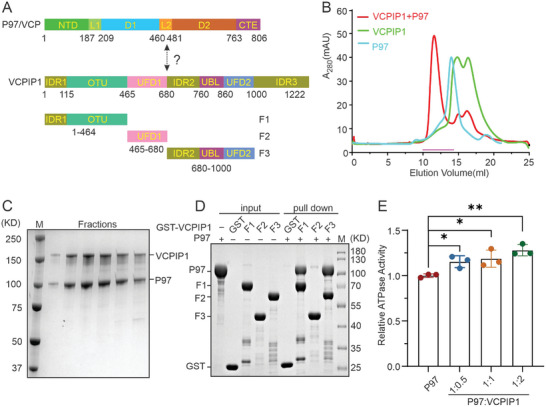
Biochemical characterization of the P97/VCP‐VCPIP1 interaction. A) Domain organization of P97/VCP and VCPIP1. NTD: N‐terminal domain; L1 and L2: Linker region 1 and 2; D1 and D2: two ATPase domains; CTE: C‐terminal extension; IDR1, 2, 3: intrinsic disordered region; OTU: deubiquitinase; UFD1 and 2: unknown function domains; UBL: ubiquitin like domain; F1, F2 and F3: truncated VCPIP1 fragments. B) Gel filtration analysis of the direct interaction between full‐length P97/VCP and VCPIP1. The fractions used for SDS‐PAGE analysis were indicated by the magenta line. C) The SDS‐PAGE combined Coomassie Brilliant Blue staining showing the protein complex fractions in panel (B). D) GST‐pulldown assays to analyze the binding of the full‐length P97/VCP and VCPIP1 fragments. E) Relative ATPase activities of P97/VCP and P97/VCP‐VCPIP1 complex. The activities are normalized to that of P97/VCP only. The data is represented as Mean ± SD (*n* = 3 independent experiments). The *p*‐values were calculated using an unpaired Student's *t*‐test in Prism software (^*^ *p* < 0.05, ^**^ *p* < 0.01, ^***^ *p* < 0.001).

Besides the N‐terminal OTU domain, VCPIP1 contained three intrinsic disorder regions (IDRs), two unknown function domains (UFDs), and a ubiquitin‐like domain (UBL) participating in diverse cellular functions (**Figure** **1**A). To validate the direct interaction between VCPIP1 and P97/VCP in vitro, we first purified the full‐length proteins and analyzed their interactions by size exclusion chromatography. The obtained result showed that the VCPIP1 could directly bind to P97/VCP by migrating together with P97/VCP on the size exclusion column (Figure 1B). A previous study identified that the C‐terminal UBL domain of VCPIP1 is responsible for P97/VCP binding and implied that VCPIP1 competes with the adaptor protein P47 for P97/VCP binding by the similar UBL domain.^[^ ^15^ ^]^ To further narrow down the P97/VCP‐binding regions in VCPIP1, we truncated VCPIP1 into three fragments, F1 (residues 1–465), F2 (residues 465–680), F3 (residues 680–1000), which contained IDR1‐OTU, UFD1 and IDR2‐UBL‐UFD2 domains, respectively (Figure 1A). We next purified the recombinant proteins and analyzed their interactions using GST‐pull down assay. Interestingly, all three VCPIP1 fragments were proved to be individually sufficient for P97/VCP binding, while the F1 and F3 fragments exhibited pretty strong binding to P97/VCP, and the binding between F2 and P97/VCP was much weaker (Figure 1D). To investigate the regulatory effect of VCPIP1 binding on the unfoldase activity of P97/VCP, we conducted an in vitro ATPase assay. The results reveal that, in the absence of substrates, the binding of VCPIP1 to P97/VCP led to a marginal increase in its activity (Figure 1E).

### 2.2 Cryo‐EM Structure of VCPIP1‐P97/VCP Complex

To elucidate the structure basis of the VCPIP1 and P97/VCP interaction, we reconstituted the complex by size exclusion chromatography and determined the structure of the VCPIP1‐P97/VCP complex using single‐particle Cryo‐EM. We collected 2202 movies for this complex on a Titan Krios 300 kV microscope and 173360 initial particles were extracted from these movies (Figure S1A–C, Supporting Information). After several rounds of 2D and 3D classifications, only one conformer was obtained and refined to high‐resolution 3D reconstruction from the dataset, and ≈97% of initial particles were used for the final 3D reconstruction and further refinement. We finally obtained a 3D reconstruction at an overall resolution of 3.45 Å (Figure S1C–F and Table S1, Supporting Information).

**Figure 2:**
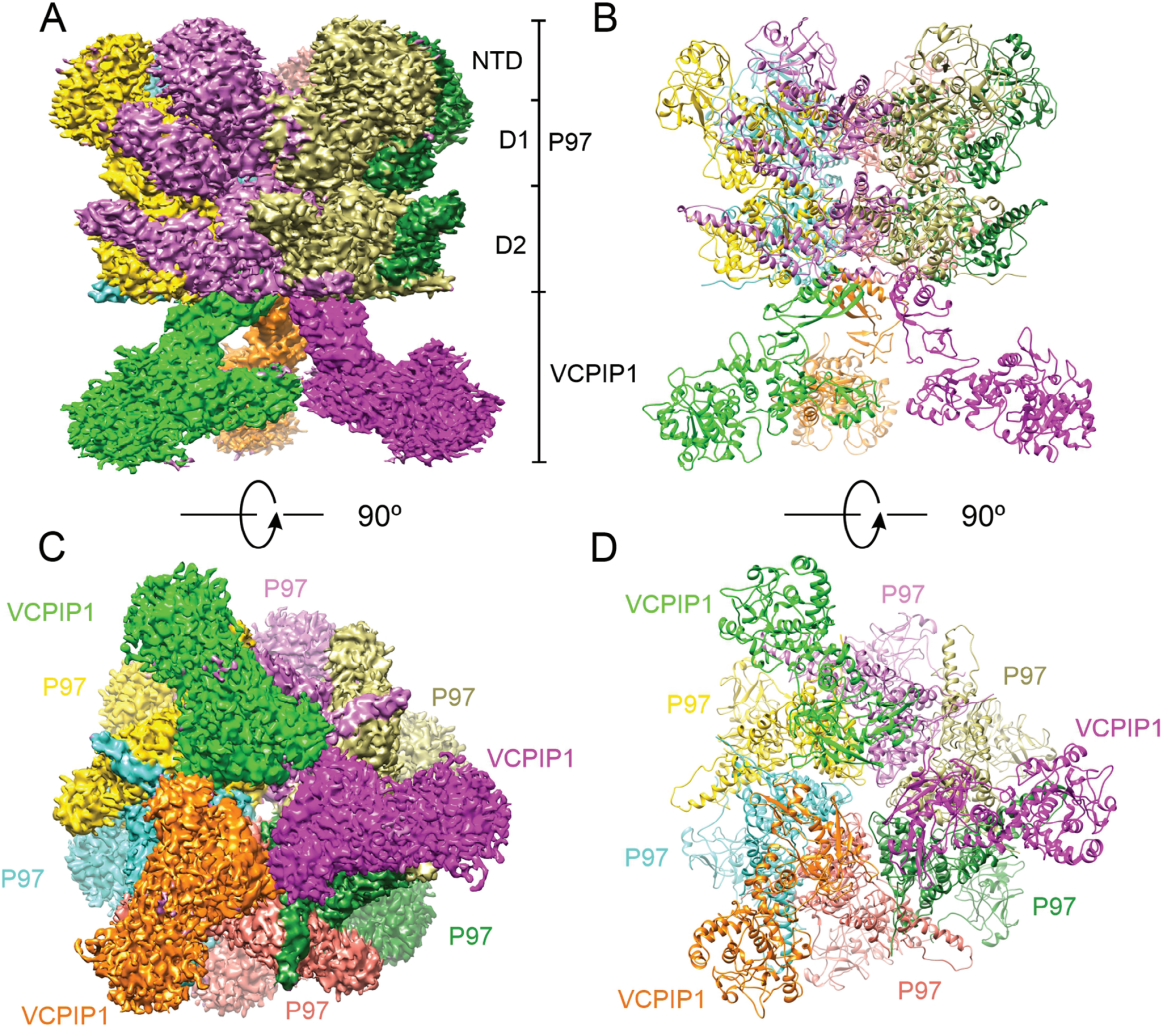
The Cryo‐EM structure of P97/VCP‐VCPIP1 complex. A) Cryo‐EM density map of P97/VCP‐VCPIP1 complex in side view. In this drawing, the NTD, D1, and D2 domains of P97/VCP hexamer and three VCPIP1 molecules were indicated. B) Ribbon diagram of the P97/VCP‐VCPIP1 complex in side view, and the protomers in this complex were colored as in panel **A**. C) Cryo‐EM density map of P97/VCP‐VCPIP1 complex in bottom view. D) Ribbon diagram of the P97/VCP‐VCPIP1 complex (bottom view).

The local resolution map reveals that the electron density of the N‐terminal domain of P97/VCP is weak, whereas it is significantly higher in the stacked rings of the D1 and D2 ATPase domains, with overall resolutions exceeding 3 Å (Figure S2A, Supporting Information). The EM density of most parts of VCPIP1 is generally low, except for the regions that directly bind to P97/VCP, suggesting that binding to P97/VCP might rigidify and stabilize VCPIP1(**Figure** **2**A; Figure S2A, Supporting Information). To build the structure of the VCPIP1‐P97/VCP complex, we docked the cryo‐EM structure of P97/VCP hexamer in ATPγS‐bound state and alpha‐fold predicted VCPIP1 structure into the cryo‐EM density map as rigid bodies, and then manually adjusted the structures to better fit the density. Albeit with the relatively lower and heterogeneous resolution of VCPIP1, the regions of VCPIP1 that directly contact P97/VCP could be readily and confidently modeled.

Expectedly, the P97/VCP in the P97/VCP‐VCPIP1 complex was folded into the canonical homo‐hexamer as previously studied (Figure 2A,C). Unexpectedly, three VCPIP1 molecules directly bind to the C‐terminal D2 ATPase domain of the P97/VCP, resulting in the formation of P97/VCP‐VCPIP1 complex with a stoichiometry of 2:1 (Figure 2B). The VCPIP1 molecules in the complex function as monomers without any contact between them (Figure 2C,D). VCPIP1 interacts simultaneously with two adjacent P97/VCP protomers through its UFD1 domain, and no other region of VCPIP1 was found to have contact with P97/VCP in our structure, in contrast to the previous prediction which suggested the C‐terminal UBL domain of VCPIP1 is responsible for P97/VCP binding. The partial UFD1 domain of VCPIP1 observed in the cryo‐EM structure folded into 4 β‐strands and 2 α‐helices (Figure 2B,D). Dali's search in PDB for this UFD1 domain didn't hit any structure with similar folding, suggesting its novel structure and unknown function. It is noteworthy that the complex structure seems to be discrepant with our biochemical data, as the F1 and F3 fragments exhibited strong binding in the GST pull‐down assay, yet no stable contact was observed in the structure of the full‐length complex (Figure 1D). The missing interaction between the F1 and F3 fragments of VCPIP1 and P97/VCP may be due to the poor cryo‐EM density, or that the structure we obtained is in the final stable state with the lowest energy. Since only one 3D reconstruction was obtained and could be refined to high resolution from our cryo‐EM studies, we favored it as the stable and dominant conformation adopted by the full‐length P97/VCP‐VCPIP1 complex (Figure S1C, Supporting Information). Although P97/VCP contains two ATPase domains, D2 is the major domain responsible for ATP hydrolysis and critical for substrate unfolding.^[^ ^19^ ^]^ The direct binding of VCPIP1 to the P97/VCP D2 domain implies that VCPIP1 may regulate the unfoldase activity of P97/VCP. However, the ATPase assay revealed that VCPIP1 binding to P97/VCP only slightly increases its ATPase activity (Figure 1E).

### 2.3 The Interface Analysis and Validation of the VCPIP1 and P97/VCP Interaction

**Figure 3:**
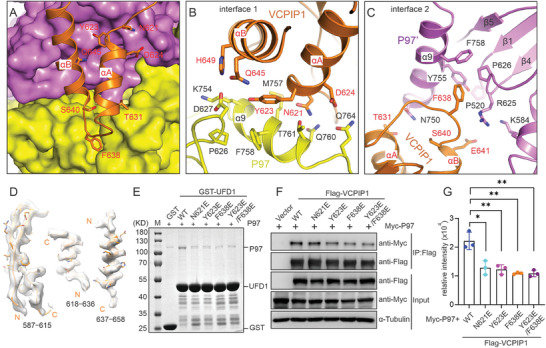
The structural analysis and validation of the P97/VCP‐VCPIP1 complex. A) The combined surface representation and the ribbon‐stick model show the interaction interface between the VCPIP1 UFD1 domain and P97/VCP hexamer. In this presentation, the P97/VCP molecules are shown in the surface model and VCPIP1 in the ribbon‐stick model. B‐C Close‐up views of the interfaces 1B) and 2C) between the VCPIP1 UFD1 domain and two adjacent P97/VCP protomers, with interacting residues are highlighted as sticks. D) Electron density of the UFD1 domain of VCPIP1. E) GST‐pulldown assays to analyze the binding affinities of the VCPIP1 UFD1 WT and mutant proteins to full‐length P97/VCP. F) Co‐IP assays to analyze the interaction between full‐length P97/VCP and VCPIP1 mutants in vivo. G) Quantitative analysis of the Co‐IP assays in panel (F). The data is represented as Mean ± SD (*n* = 3 independent experiments). The quantification of the band intensities was performed with the software ImageJ. The *p*‐values were calculated using an unpaired Student's *t*‐test in Prism software (^*^ *p* < 0.05, ^**^ *p* < 0.01, ^***^ *p* < 0.001).

VCPIP1 contacts P97/VCP through two major interfaces. The residues on the αA and αB of the UFD1 domain of VCPIP1 form extensive hydrophobic and hydrophilic interactions with the residues on the α9 and β4‐β5 loop of the D2 domains from two adjacent P97/VCP protomers (**Figure** **3**A). Because the electron density of the UFD1 domain is pretty clear, we can definitively assign the precise orientation and conformation of the side chains of the key residues on the interfaces (Figure 3D).

At interface 1, polar residues N621 and D624 from the αA of UFD1 constitute several hydrogen bonds with the hydrophilic side chains of the T761, Q760, and Q764 on the α9 of P97/VCP (Figure 3B). Moreover, the aromatic residue Y623 plays a pivotal role in mediating the complex formation in interface 1, the side chain of Y623 pointed into the hydrophobic pocket formed by the side chains of P626, K754, M757, and F758 from the α9 and β4‐β5 loop of the D2 domain in P97/VCP. In addition, the polar residues of Q645 and H649 from the αB of the UFD1 form hydrogen bonds with the side chains from D627 and K754 of the D2 domain (Figure 3B). At interface 2, F638 on the αA–αB loop of UFD1 plays a central role in maintaining the interaction. The aromatic side chain of F638 inserts into a hydrophobic pocket formed by residues R625, P626, Y755, and F758 from the β4‐β5 loop and α9 of the D2 domain, resulting in extensive hydrophobic interactions (Figure 3C). The negatively charged residue E641 of VCPIP1 is located in close proximity to the positively charged residues R625 and K584 from P97/VCP. These residues are likely to develop favorable electrostatic and hydrophobic interactions. Furthermore, two hydrophilic residues, T631 and S640, of VCPIP1 form two additional hydrogen bonds with N750 on the α9 of P97/VCP, further strengthening the complex (Figure 3C).

To validate the contacting interfaces, we mutated the key residues on the interfaces of VCPIP1 and purified the GST‐tagged proteins. The subsequent GST‐pull down assay confirmed the direct interaction between VCPIP1's UFD1 domain and P97/VCP and revealed that any single mutation, such as N621E, Y623E, or F638E, or the double mutation Y623E/F638E on the interface, abolished the interaction (Figure 3E). To further validate the complex structure in vivo on the full‐length proteins, we carried out the co‐immunoprecipitation (Co‐IP) assay. The results demonstrated that single mutations in VCPIP1, such as N621E, Y623E, or F638E, significantly decreased the binding ability to P97/VCP, and the double mutation Y623E/F638E further reduced the interaction (Figure 3F,G). The presence of additional interfaces between P97/VCP and VCPIP1 may explain why the VCPIP1 Y623E/F638E double mutant still exhibited weakened binding to P97/VCP.

### 2.4 Both the N‐ and C‐Terminal Regions of P97/VCP Interact with VCPIP1

**Figure 4:**
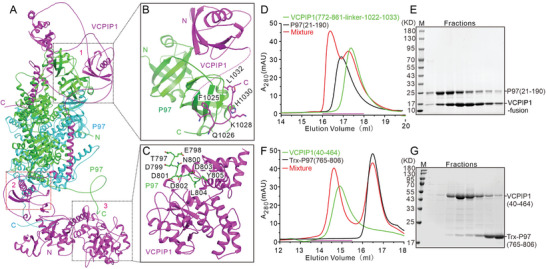
Both the N‐ and C‐terminal regions of P97/VCP are associate with VCPIP1. A) Ribbon diagram of the structural model of the P97/VCP‐VCPIP1 complex predicted by AlphaFold 3 using two P97/VCP protomers and one VCPIP1 molecule. In this drawing, the P97/VCP protomers are colored in green and cyan, while the VCPIP1 is colored in magenta. The three major interfaces between P97/VCP and VCPIP1 are indicated. B) Close‐up views of the interfaces between the P97/VCP N‐terminal domain (NTD, residues 21–190) and the VCPIP1 UBL domain (residues 770–860), as well as the subsequent motif on the IDR3 (residues 1025–1032). C) Close‐up view of the interface between the C‐terminal tail of P97/VCP and the N‐terminal region of VCPIP1. D) Gel filtration analysis of the direct interaction between the P97/VCP NTD (residues, 21–190) and a fusion protein of the VCPIP1 UBL domain (residues 772–861) linked to the short motif in IDR3 (residues 1022–1033) by a linker sequence (GGSSGGSSG). The fractions used for SDS‐PAGE analysis were indicated by the magenta line. E) SDS‐PAGE combined with Coomassie Brilliant Blue staining showing the protein complex fractions in panel (D). F) Gel filtration analysis of the direct interaction between the C‐terminal tail of P97/VCP (residues 765–806) and the N‐terminal region of VCPIP1 (residues 40–464). The fractions used for SDS‐PAGE analysis were indicated by the magenta line. (G) SDS‐PAGE combined with Coomassie Brilliant Blue staining showing the protein complex fractions in panel (F).

To further characterize the additional interfaces between VCPIP1 and P97/VCP, we predicted the complex structure comprising one VCPIP1 molecule and two P97/VCP protomers using the AlphaFold 3 server, based on the ratio observed in our cryo‐EM structure. The resulting structural model indicates that VCPIP1 binds to P97/VCP through three major interfaces, with both the N‐terminal and C‐terminal regions of P97/VCP required for VCPIP1 binding (**Figure** **4**A). Interface 2 is constituted by the VCPIP1 UFD1 and the two adjacent D2 domains of P97/VCP, as observed in our cryo‐EM structure (Figure 4A). At Interface 1, the UBL domain of VCPIP1 (residues 770–860) and the subsequent motif on IDR3 (residues 1025–1032) are suggested to interact with the N‐terminal domain (NTD) of P97/VCP (residues 21–190) (Figure 4B). To validate this interface, we created a fusion construct by directly linking the UBL domain and the short motif of VCPIP1 with a short linker (GGSSGGS) and purified the fusion protein to analyze its interaction with the P97/VCP NTD. Gel filtration and SDS‐PAGE analysis revealed that the fusion protein of VCPIP1 co‐migrates with the P97/VCP NTD on the size exclusion column, indicating a direct interaction between the two proteins (Figure 4D,E). The C‐terminal tail of P97/VCP makes close contact with the N‐terminal region of VCPIP1, forming the third interface (Figure 4C). This interaction was also confirmed by gel filtration co‐migration experiments (Figure 4F,G). Additionally, our cryo‐EM map shows an unaccounted, continuous density connecting the last modeled C‐terminal residue of P97/VCP to the N‐terminal OTU domain of VCPIP1. This weak density likely belongs to the C‐terminal tail of P97/VCP, though the map quality in this region does not allow us to model the atomic structure of the complete tail of P97/VCP (Figure S3, Supporting Information). Overall, the analysis demonstrates that the P97/VCP tail binds to the OTU domain of VCPIP1, while the NTD of P97/VCP simultaneously contacts the UBL domain and the IDR3 short motif of VCPIP1. Given the poor electron densities of the P97/VCP NTDs domain and the VCPIP1 OTU domain due to their dynamic nature, the interactions between the F1 and F3 fragments of VCPIP1 and P97/VCP are unobserved in the cryo‐EM structure.

### 2.5 VCPIP1 Binding Promotes P97/VCP into NTDs “UP” State

**Figure 5:**
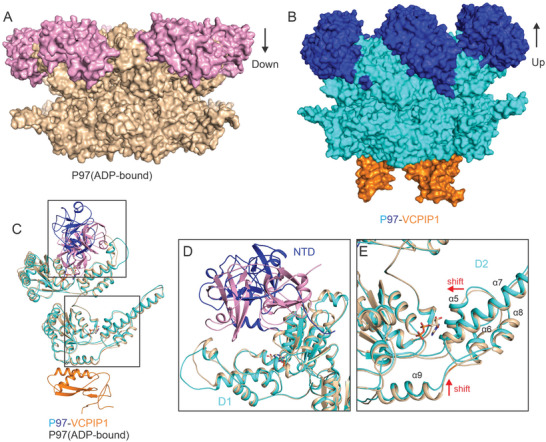
VCPIP1 binding promotes P97/VCP in an active state. A) Surface view of P97/VCP hexamer (PDB:7vct) in ADP‐bound form (inactive state). B) Surface view of P97/VCP‐VCPIP1 complex (active state). C) Superimposed ribbon diagrams of the P97/VCP in ADP bound form and P97/VCP in complex with VCPIP1. D) Close‐up view of the NTD‐D1 domains of P97/VCP in the superimposed structures of the P97/VCP in ADP bound form and P97/VCP‐VCPIP1 complex. E) Close‐up view of the D2 domains of P97/VCP in the superimposed structures of the P97/VCP in ADP bound form and P97/VCP‐VCPIP1 complex.

Previous structural studies have reported that the N‐terminal domains of the P97/VCP hexamer adopt an “UP” conformation when bound to ATP, and a “down” conformation when ATP is hydrolyzed to ADP^[^ ^19^, ^29^ ^]^ (**Figure** **5**A). However, in the VCPIP1‐P97/VCP complex structure, all P97/VCP molecules are in “UP” conformation without ATP being added to the solution (Figure 5B). Furthermore, no nucleotide density was observed in the nucleotide‐binding pockets of both the D1 and D2 ATPase domains of P97/VCP. Given that the recombinant P97/VCP purified from *E. coli* would be prebound to ADP, the binding of VCPIP1 may facilitate the releasing of ADP from the P97/VCP, thereby preparing it for subsequent ATP binding.^[^ ^29^ ^]^ The apo (nucleotide‐free) status of the P97/VCP dodecamer structure exhibits NTDs “UP” conformation, resembling the conformation observed in the ATP‐bound P97/VCP hexamer.^[^ ^30^, ^31^ ^]^ Our structure indicates that the binding of VCPIP1 to the D2 domain of P97/VCP would induce a conformational change, promoting P97/VCP into the NTDs “UP” state. Indeed, the overlay structure of P97/VCP in the ADP‐bound state and P97/VCP in the complex has confirmed a significant conformational change in P97/VCP upon binding to VCPIP1, with an RMSD of 1.03 Å. This conformational change mainly occurs in the N‐terminal and D2 domains, while the D1 domains show no obvious differences (Figure 5C,D). The close‐up view of the D2 domain of overlaid structures reveals that a major inward shift occurs in α5‐α7 around the nucleotide‐binding pocket when VCPIP1 directly binds to the D2 domain (Figure 5D). This inward shift in the D2 domain is responsible for the overall structural variation, thereby promoting its transition into the “UP” state and finally affecting its unfoldase function.

### 2.6 VCPIP1 Promotes P97/VCP Binding to SNARE Substrates for Golgi Fusion

VCPIP1 residues that lie at the two VCPIP1‐P97/VCP interfaces are all conserved from different species, suggesting that they might be functionally important (Figure S4A, Supporting Information). Previous studies have proved that VCPIP1 interaction with P97/VCP synergistically facilitates the post‐mitotic Golgi vesicle fusion by regulating the priming of syntaxin‐5 containing SNARE complex through the P97/VCP adaptor protein P47 during the cell cycle.^[^ ^15^ ^]^ To explore the functional relevance of these interfaces in regulating the SNARE complex, we first purified the substrate SNARE complex. The SNARE complex, composed of t‐SNARE protein syntaxin‐5 (STX‐5) and v‐SNARE protein Bet1, is responsible for the post‐mitotic Golgi vesicle fusion. However, the other two t‐SNARE proteins that make up this complex have not been identified yet. So, we switched to choosing the SNARE complex, which also contains syntaxin‐5 and Bet1 proteins in charge of vesicle fusion from the endoplasmic reticulum to the smooth Golgi apparatus, as the mimic substrate.^[^ ^32^, ^33^ ^]^ The gel filtration chromatography combined with SDS‐PAGE results reveals that the separately purified SNARE proteins without the transmembrane domain, containing syntaxin‐5, GOSR1, Ykt6, and Bet1, migrate together to constitute the intact SNARE complex in vitro (Figure S5A, Supporting Information).

**Figure 6:**
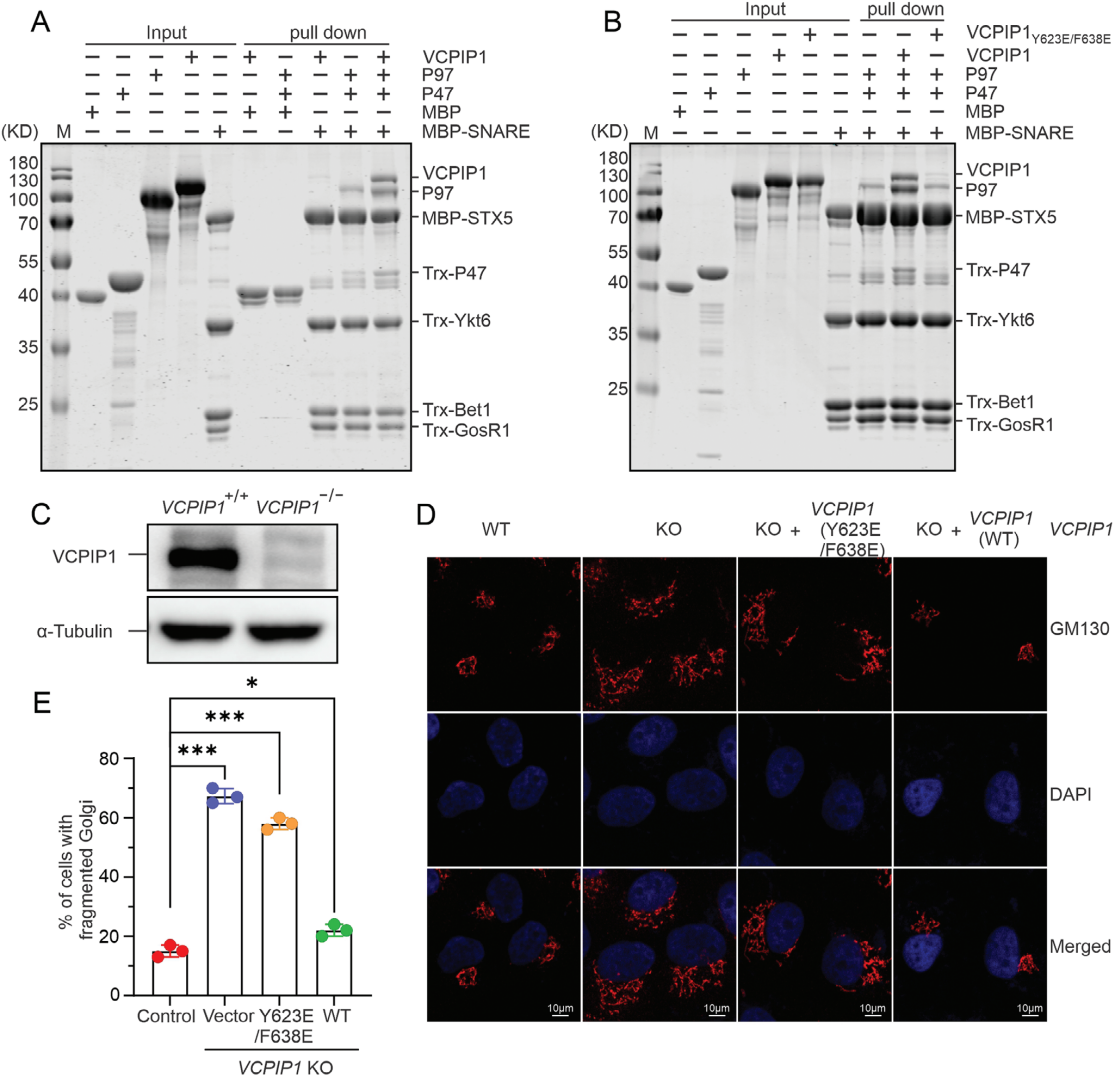
The direct binding of VCPIP1 to P97/VCP is required for the post‐mitotic Golgi fusion. A) MBP pull‐down assays to analyze the binding affinities of Syntaxin5(STX5) contained SNARE complex to the VCPIP1, P97/VCP‐P47 and VCPIP1‐P97/VCP‐P47 complex. B) MBP pull‐down assays to analyze the binding of Syntaxin5(STX5) contained SNARE complex to the P97/VCP‐P47, or in complex with VCPIP1 WT or Y623E/F638E double mutant. C) Western blot analyses of VCPIP1 expression in the wild‐type and *VCPIP1* knock‐out Hela cells generated by the CRISPR/Cas9 system. The anti‐tubulin analysis is used as the input assay. D) Cell immunofluorescence assays stained by Golgi marker GM130 antibody to show the Golgi morphology in Hela cells of wide‐type, *VCPIP1*‐KO and *VCPIP1*‐KO rescued by *VCPIP1* WT or Y623E/F638E mutant plasmids. The nucleus is shown by staining DAPI. E) Statistical results of the cells with fragmented Golgi related to the panel (D). The data represented as Mean ± SD of 20 analyzed cells from three independent experiments (*n* = 3). The *p*‐values were calculated using unpaired Student's *t*‐test in Prism software (^*^ *p* < 0.05, ^**^ *p* < 0.01, ^***^ *p* < 0.001).

MBP pull‐down assay confirmed that P97/VCP can form a stable complex with the syntaxin‐5‐contained SNARE complex through the adaptor protein P47. To our surprise, adding VCPIP1 protein to the reaction system largely enhanced the binding of the P97/VCP‐P47 complex to the SNARE substrate (**Figure** **6**A). However, compared with the VCPIP1 WT protein, the Y623E/F638E double mutant protein which has a much weaker interaction between VCPIP1 UFD1 and P97/VCP, did not promote the binding of P97/VCP‐P47 to the SNARE substrate (Figure 6B). These biochemical assays further demonstrated that VCPIP1 contacts the D2 domain of P97/VCP through its UFD1 domain, inducing a conformational change in P97/VCP to favor its binding to the SNARE substrate, thereby regulating the disassembly of the SNARE complex.

To further investigate the functional relevance of the P97/VCP‐VCPIP1 complex in post‐mitotic Golgi reassembly, we generated a VCPIP1‐knockout HeLa cell line using CRISPR‐Cas9 technology. Immunoblotting results with a specific antibody recognizing VCPIP1 confirmed the successful knockout of endogenous VCPIP1(Figure 6C). Cell immunofluorescence experiments were performed using the Golgi marker GM130 antibody to visualize Golgi morphology. As expected, VCPIP1 depletion resulted in dramatic Golgi fragmentation, whereas the transfection with control guide RNA had no effect (Figure 6D). In order to confirm the functions of VCPIP1 in the post‐mitotic Golgi membrane fusion, we transfected the WT and mutant VCPIP1 plasmids into the VCPIP1 KO cells and then analyzed the Golgi morphology by GM130 staining. The immunofluorescence results revealed that re‐expressing of the WT VCPIP1 in the knockout cell rescued the Golgi fragmentation, while the expression of P97/VCP binding‐deficient VCPIP1 Y623E/F638E mutant didn't rescue the fragmentation of Golgi (Figure 6D,E). These data revealed that VCPIP1 UFD1 interaction with P97/VCP is strictly required for post‐mitotic Golgi fusion, which is probably due to the enhanced binding ability of the P97/VCP‐P47 complex to the SNARE substrates by VCPIP1 UFD1 binding, ultimately promoting membrane fusion.

Based on our results, we proposed a model to illustrate the functions of the VCPIP1‐P97/VCP‐P47 complex in the post‐mitotic Golgi reassembly (Figure S6, Supporting Information). During the late telophase, Golgi tubulovesicles undergo a remarkable congregation in the pericentriolar region, initiating the reconstitution of a functional Golgi through a distinctive fusion process. In this process, a Golgi vesicle harboring Syntaxin‐5 (v‐SNARE) approaches a target vesicle containing the t‐SNARE protein Bet1. Subsequently, the two vesicles merge, forming a larger vesicle facilitated by the assembly of the intact SNARE complex, assisted by two additional t‐SNARE proteins. Then, the tightly assembled SNARE complex can be primed by either the unfoldase machinery VCPIP1‐P97/VCP‐P47 or the NSF‐SNAPs complex, leading to the release of the SNARE domain of Syntaxin‐5. This crucial step allows for the subsequent fusion of the Golgi vesicle, enabling it to undergo a second round of fusion and regenerate the Golgi cisterna. The assembly of the Golgi cisterna into a functional Golgi structure occurs through a series of stacking and linking processes.

## 3 Discussion

The Golgi apparatus is a critical membranous organelle consisting of a series of stacked flattened cisternae to process and package proteins and lipid molecules toward their different downstream locations (1‐2). Like the endoplasmic reticulum (ER), the Golgi apparatus is systematically remodeled once committed to mitotic entry (2‐5). The substantial morphological remodeling of the Golgi after mitosis is regulated by P97/VCP‐VCPIP1 mediated Golgi membrane fusion (11, 15, 22, 24). The failure in the structural reassembly of the Golgi membrane would result in its fragmentation with functional disorder in protein glycosylation, localization, and degradation (3, 4). Yet, how P97/VCP‐VCPIP1 organizes and promotes Golgi membrane remodeling, has not been well clarified. In this study, our structural and functional studies of the P97/VCP‐VCPIP1 complex provide key insights into the organization and membrane fusion mechanism of the underappreciated P97/VCP‐VCPIP1 complex.

VCPIP1 was initially identified as an essential factor for P97/VCP/p47‐mediated Golgi and ER assembly (15). It was shown to bind to the P97/VCP/p47/syntaxin5 complex and dissociate it via P97/VCP‐catalyzed ATP hydrolysis.^[^ ^15^ ^]^ Later, although with unknown ubiquitinated substrates, VCPIP1 was reported to act as a deubiquitinating enzyme during P97/VCP/p47‐mediated reassembly of mitotic Golgi fragments.^[^ ^24^ ^]^ Furthermore, a Golgi protein WAC was specifically identified as a novel VCPIP1‐binding protein in the Golgi membrane. Its binding promotes VCPIP1's deubiquitinating activity and is required for P97/VCP/p47‐mediated Golgi reassembly, but not for P97/VCP/p37‐mediated reassembly.^[^ ^22^ ^]^ Consistently, our studies have demonstrated that VCPIP1 biochemically promotes P97/VCP‐p47 binding to SNARE substrates, the addition and binding of WAC protein to VCPIP1 might further stimulate their binding for membrane fusion.

Recently, VCPIP1 has been reported to associate with both the N‐ and C‐terminal regions of P97/VCP via its C‐ and N‐terminal regions, respectively.^[^ ^28^ ^]^ Our biochemical binding assays also showed that multiple individual domains (including IDR1‐OTU, UFD1, and IDR2‐UBL‐UFD2) from VCPIP1 are sufficient for binding to full‐length P97/VCP, among which UFD1 acts as the weakest binder. The weak binding between P97/VCP and VCPIP1 UFD1 region, suggests that the UFD1 itself might not fold properly for P97/VCP binding in vitro. The unique folding of UFD1 with four β‐strands followed by two α‐helices might be the underlying reason for its difficulty in proper folding. In contrast to the binding assays using individual domains, the incubation of both full‐length P97/VCP and VCPIP1 for Cryo‐EM studies results in a stable complex with the UFD1 as the only contacting site. It is not uncommon that the protein‐protein interaction in the full‐length proteins may not be consistent with the isolated domains. We favor that the structure of full‐length P97/VCP‐VCPIP1 we obtained by Cryo‐EM is the dominant and final stable state with the lowest energy. The Y623 and F638 sites from UFD1 contribute to the major binding, which is also confirmed by our cellular Golgi membrane fusion assay. We concluded that this UFD1‐mediated VCPIP1 interaction with P97/VCP is critical for their function in Golgi reassembly. How the multiple sites from VCPIP1 coordinate for binding to P97/VCP and contribute to Golgi membrane fusion in vitro and in vivo is an interesting question worth further investigation.

Our structural analysis reveals that three individual VCPIP1 molecules sit over the C‐terminal proposed substrate exit tunnel of P97/VCP homo‐hexamer, resulting in a consecutive C6 to C3 symmetric barrel‐like structure. Clearly, the saturated binding of P97/VCP by VCPIP1 results in this unique architecture for this fascinating molecular motor. In this binding mode, the three VCPIP1 molecules residing at the C‐terminal substrate exit tunnel of P97/VCP might be able to deubiquitinate the ubiquitinated substrates with very high efficiency once they are unfolded and released by P97/VCP unfoldase. The UFD1 domain from VCPIP1 docks to the two neighboring D2 domains of P97/VCP, which might become more rigidified for substrate unfolding. The stabilized D2 would further allosterically regulate the NTDs of P97/VCP in a “UP” conformation for binding of cofactors, like P47 and Npl4‐Ufd1.

The inheritance of the Golgi apparatus into the daughter cells during each cell division cycle is mediated by a disassembly‐to‐reassembly process, which is tightly controlled by phosphorylation and ubiquitination.^[^ ^6^, ^25^ ^]^ In early mitosis, phosphorylation of the VCIP135 S130 site by Cdk1, has been shown to be sufficient to inactivate its deubiquitinase activity and inhibit P97/VCP/p47‐mediated Golgi membrane fusion.^[^ ^26^ ^]^ At the end of mitosis, VCIP135 S130 is dephosphorylated, and its enzymatic activity recovers for Golgi reassembly.^[^ ^26^ ^]^ By structural analysis of the P97/VCP‐VCPIP1 complex, this site S130 is located in the N‐terminal OTU domain and clearly not located within the UFD1 for VCP interaction. Whether the interaction between P97/VCP and VCPIP1 is directly regulated by CDK1 or other mitotic kinases needs further exploration. The VCPIP1 UFD1 has been identified to harbor multiple ubiquitination sites, such as K571, K632, K646, K657, and K658.^[^ ^34^ ^]^ Considering the key role in contact with VCP by VCPIP1 Y623 and F638, which are the neighboring residues of K632 and K646, UFD1 ubiquitination might have a direct effect on their interaction and thus regulate Golgi membrane dynamics.

In conclusion, our findings uncover the high‐resolution structure of the P97/VCP‐VCPIP1 complex and reveal the unique and unexpected binding modes between the ATP‐driven substrate unfoldase P97/VCP and the deubiquitinase VCPIP1. Our work demonstrates that the UFD1 of VCPIP1‐mediated interaction with P97/VCP is strictly required for Golgi membrane reassembly. Our studies provide novel views on the fascinating and multifaced molecular motor P97/VCP in the quality control of organelles, like the Golgi apparatus.

## 4 Conclusion

We solved the cryo‐EM structure of the VCPIP1‐P97/VCP complex and revealed that three individual VCPIP1 molecules directly bind to the C‐terminal D2 domains of the P97/VCP hexamer through their UFD1 domains. The N‐ and C‐terminal regions of P97/VCP are both required for its association with VCPIP1. The P97/VCP C‐terminal tail was found to bind to the N‐terminal OTU domain (residues 40–464) of VCPIP1, while the NTD (residues 21–190) of P97/VCP simultaneously contacts the UBL domain (residues 770–860) and the IDR3 motif (residues 1025–1032) of VCPIP1. In this complex, P97/VCP is in the apo (nucleotide‐free) state with its N‐terminal domains in the “UP” conformation. The binding of VCPIP1 may facilitate the release of ADP from the P97/VCP complex, thereby priming it for subsequent ATP binding. The interfaces between VCPIP1 UFD1 and P97/VCP have proved to be indispensable for the post‐mitotic Golgi reassembly.

## 5 Experimental Section

### Antibodies

The following antibodies against human proteins were used for immunoblotting and immunofluorescence: anti‐Myc (Cell Signaling Technology, PA1‐981), anti‐Flag (Sigma‐Aldrich, F1804), anti‐GM130 (Cell Signaling Technology, 12 480), anti‐α‐tubulin (Sigma‐Aldrich, T6199‐100UL), and anti‐VCPIP1 (ABclonal, A16572). The HRP‐conjugated secondary antibodies used for immunoblotting were purchased from Jackson ImmunoResearch.

### Protein Expression and Purification

The full‐length gene of human VCPIP1, P97/VCP, P47, Syntaxin5, GosR1, Ykt6 and Bet1 were synthesized from Tsingke company. The different complementary DNA fragments encoding the full‐length human VCPIP1, P97/VCP and P47 were PCR amplified and cloned into the pRSF‐32 m vector (a modified version of the pRSF‐Duet vector that introduces N‐terminal thioredoxin and 6xHis tags before the first multiple cloning site)^[^ ^35^ ^]^ or pET‐28a vector for the subsequent recombinant protein expression or cloned into the PCS2‐Flag or PCS2‐Myc vector for the Co‐IP and the immunofluorescence experiments, respectively. For the GST‐pull down assay, the DNA fragments encoding the human VCPIP1 (residues 1–464, 465–680, and 680–1000) were amplified by PCR and cloned into the pGEX‐6P‐1 vector for expression of GST‐tagged recombinant protein. To make the intact SNARE complex, the cDNAs encoding the SNARE proteins, containing syntaxin5 (residues 55–330), GosR1 (residues 150–230), Ykt6 (residues 1–193) and Bet1 (residues 1–93) were PCR amplified and cloned into the pET‐MBP vector (a modified version of the pET‐32a vector that introduces N‐terminal Maltose Binding Protein before the first multiple cloning site) or pRSF‐32 m vector for the intact SNARE complex protein expression. All the plasmids used in this study were constructed using a Seamless Assembly Kit and performed according to the manufacturer's protocol (ABclonal, RK21020). All the point mutations of VCPIP1 used in this study were created using the standard PCR‐based mutagenesis method, further checked by DNA sequencing.

The recombinant proteins were expressed in BL21 (DE3) *Escherichia coli* bacterial cells. The bacterial cultures were grown in an LB medium at 37 °C. When the OD_600_ of the culture reached 0.8, the temperature was lowered to 16 °C, and then the protein expression was induced by adding 200 µm IPTG, followed by further incubation at 16 °C overnight.

For purification of the full‐length P97/VCP, VCPIP1, and P47 proteins, the bacterial cell pellets were collected and then resuspended in five volumes of the binding buffer (50 mm Tris‐HCl, pH 7.9, 500 mm NaCl, 5 mm imidazole). Subsequently, the cells were lysed by the ultrahigh‐pressure homogenizer machine ATS‐1500 (ATS Engineering Limited). The resulting cell lysate was spun down by centrifuge at 35000 g and 4 °C for 30 min to remove the pellets. The supernatant was carefully transferred into a new 50‐mL centrifuge tube and mixed with Ni^2+^‐NTA agarose resin (Qiagen, 30 230) that had been pre‐equilibrated by the binding buffer. The mixture was incubated at 4 °C for 1 h with rotation. After extensive washing with wash buffer (50 mm Tris‐HCl, pH 7.9, 500 mm NaCl, 30 mm imidazole), the His_6_‐tagged proteins were eluted from the Ni^2+^‐NTA resin by the elution buffer (50 mm Tris‐HCl, pH 7.9, 500 mm NaCl, 400 mm imidazole). The target proteins were further purified by size exclusion chromatography with the UNIONDEX 200PG column (Union Biotech). The VCPIP1‐P97/VCP complex was made by directly mixing the two purified proteins at a 1:1 molar ratio and then incubated on ice for 1 h. The mixture was separated by the Superose 6 Increase10/300GL size exclusion column (Cytiva). The pooled peak fractions were combined for concentration and then stored at −80 °C for future use.

To purify the intact SNARE complex, the individual SNARE proteins, containing MBP‐tagged syntaxin5 (residues 55–330) and His_6_‐tagged GosR1 (residues 150–230), Ykt6 (residues 1–193) and Bet1 (residues 1–93) proteins were separately expressed in BL21 (DE3) *Escherichia coli* bacterial cells. The pellets of 1 L cell cultures for each protein were collected by centrifugation and resuspended in five volumes of buffer A (50 mm Tris‐HCl, pH 7.5, 100 mm NaCl, 1 mm DTT). The resuspended pellets were mixed and lysed by the ultrahigh‐pressure homogenizer. The lysate was cleared by centrifugation at 35000 g and 4 °C for 30 min. The supernatant was transferred into a new 50 mL centrifuge tube and mixed with amylose resin (NEB, E8021L) that had been pre‐equilibrated by buffer A. The mixture was incubated at 4 °C for 1 h with rotation. The target protein was eluted from the resin by 10 mm D‐Maltose dissolved in buffer A after extensive wash with buffer A. The complex protein was further purified by the UNIONDEX 200PG column (Union Biotech). Fractions containing the intact SNARE complex were pooled, concentrated, and stored at −80 °C.

For purification of GST‐tagged protein, cell pellets were resuspended in PBS buffer and lysed by the ultrahigh‐pressure homogenizer. The cleared supernatant was mixed with appropriate pre‐equilibrated glutathione Sepharose 4B resin (GE Healthcare). The mixture was incubated at 4 °C for 1 h with rotation. After extensive washing with PBS, the target protein was eluted by the elution buffer (50 mm Tris‐HCl, pH 7.5, 500 mm NaCl, 15 mm reduced L‐glutathione). The eluted protein was further purified by the UNIONDEX 200PG column (Union Biotech). Fractions containing the target proteins were pooled, concentrated, and stored at −80 °C.

### Cryo‐EM Data Collection and Image Processing

For cryo‐EM grid preparation, 3 µL VCPIP1–P97/VCP complex samples (≈10 mg mL^−1^) were applied onto glow‐discharged holey carbon grids (Quantifoil Cu R1.2/1.3, 300 mesh), blotted with a Vitrobot Marker IV (Thermo Fisher Scientific) for 3 s under 100% humidity at 4 °C, and subjected to plunge freezing into liquid ethane. All cryo‐EM data were collected using the FEI Titan Krios microscope at 300 kV equipped with a Gatan K3 Summit direct electron detector (super‐resolution mode, at a nominal magnification of 81000) and a GIF‐quantum energy filter. Defocus values were set from −1.0 to −2.0 µm. Each stack of 32 frames was exposed for 1.56 s, with a total electron dose of 50 e‐/Å2. AutoEMation was used for fully automated data collection.

All micrograph stacks were motion‐corrected with MotionCor2^[^ ^36^ ^]^ with a binning factor of 2, resulting in a pixel size of 1.0773 Å. Contrast transfer function (CTF) parameters were estimated using Gctf. Most steps of image processing were performed using cryoSPARC.^[^ ^37^ ^]^ For 3D processing of the OGT data, a total of 173360 particles were automatically picked from 2202 micrographs using Gautomatch (developed by Kai Zhang, MRC‐LMB). Particles were extracted with a pixel size of 4.3092 Å and subjected to several rounds of reference‐free 2D classification. 173360 particles were kept after excluding obvious ice contamination and junk particles and reextracted without binning. Then, ab initio models were generated and subsequently used for heterogeneous 3D refinement. The best class of 168188 particles was then used for further non‐uniform refinement and local refinement and was used for further structural analysis. The global resolution of the VCPIP1–P97/VCP is 3.45 Å based on the Fourier Shell Correlation (FSC) 0.143 criterion.

### Model Building and Refinement

The structures of human full‐length P97/VCP (PDB: 7VCV) and VCPIP1 (Alphafold: AF‐P55072)^[^ ^38^ ^]^ were used as the starting models and docked into the final Cryo‐EM maps with UCSF Chimera.^[^ ^39^ ^]^ The models were manually adjusted and iteratively built in COOT^[^ ^40^ ^]^ and then refined against summed maps using Phenix.real_space_refine implemented in PHENIX^[^ ^41^, ^42^ ^]^ until the validation data were reasonable. FSC values were calculated between the resulting models and the two half‐maps, as well as the averaged map of the two half‐maps. The quality of the models was evaluated with MolProbity^[^ ^43^, ^44^ ^]^ and EMRinger. The structure validation statistics are listed in Table S1 (Supporting Information). All structural figures were prepared with PyMOL (Schrodinger), Chimera, or Chimera X.^[^ ^39^ ^]^

### GST‐Pull Down Assay

The in vitro binding between P97/VCP and GST‐tagged VCPIP1 proteins was analyzed in the reaction buffer containing 20 mm Tris (pH 7.5), 100 mm NaCl, and 1 mm DTT. 50 µg of different GST‐tagged proteins were mixed with full‐length P97/VCP protein at the molar ratio 1:3 in 200 µL reaction buffer. 20 µL pre‐equilibrated glutathione Sepharose 4B resin (GE Healthcare) was applied to each reaction mixture to pellet the P97/VCP and GST‐VCPIP1 complexes. The pellets were washed four times with the reaction buffer and then boiled with 2X SDS–PAGE loading buffer. The proteins were separated by SDS‐PAGE and visualized by Coomassive‐blue staining.

### ATPase Assay

The ATPase reactions were conducted using the ADP‐Glo Kinase Assay kit (Promega) in white OptiPlate‐384 plates (Perkin Elmer) at room temperature. To initiate the reactions, P97/VCP and VCPIP proteins were diluted in the Reaction Buffer (25 mm Tris‐HCl pH 7.5, 100 mm NaCl, 20 mm MgCl2, 1 mm DTT) and incubated together at room temperature for 30 min at the desired molar ratio. ATP was then added to a final concentration of 0.2 mm in each reaction, with a final volume of 10 µL, and incubated for 60 min. The final monomer concentration of P97/VCP was 600 nm. After the incubation, 10 µL of the ADP‐Glo Reagent was added to each reaction and incubated for an additional 30 min at room temperature. Subsequently, 20 µL of the detected reagent from the kit was added to the reaction and incubated for 30 min. The luminescence was measured using the VICTOR Nivo Plate Reader (Perkin Elmer). All data were normalized to the reading of the sample containing only the Reaction Buffer in the presence of ATP without any proteins. The ATPase assays were repeated three times. Relative ATPase activities of the P97/VCP‐VCPIP complex were determined at the indicated molar ratios of 1:0.5, 1:1, and 1:2. The activities were normalized to the P97/VCP‐only group. The data are presented as means ± SD (*n* = 3 independent experiments).

### Cell Culture, Transfection, and Knockout Cell Line Generation

HeLa cells were grown in DMEM (Gibco, 11 995 065) supplemented with 10% fetal bovine serum (FBS, Sigma, F8318). The cell line was regularly checked by DAPI staining to exclude the mycoplasma contamination. All the plasmids used for mammalian expression in this study were derived from the modified pCS2 vector with N‐terminal Myc6 or 3X Flag tags. Plasmid transfection was performed using the Lipofectamine 2000 Transfection Reagent (Thermo Fisher Scientific, 11 668 019) according to the manufacturer's protocols.

The VCPIP1 gene was knocked out from HeLa cells using the CRISPR/Cas9 system, with a guide RNA spanning the exon 1. Guide RNA sequence: 5′‐GTGTAGCACTACGTCCGGGT‐3′. The guide RNA was individually cloned into the Lenticrisprv2 vector (v2‐sgVCPIP1), v2‐sgVCPIP1 with psPAX2, and pVSVg plasmids were transfected into HEK293T cells using Lipofectamine 2000, After 48 h, the supernatant lentivirus was collected and mixed with the complete medium to culture Hela cells for 36 h, and the uninfected cells were killed by puromycin (1ug mL^−1^) for 48 h. Single colonies were screened by western blot using a specific VCPIP1 antibody (1:1000 dilution, ABclonal, A16572) to confirm the loss of VCPIP1 protein expression and detected the expression of Tubulin with anti‐Tubulin antibody as the loading control (1:5000 dilution, Sigma, SAB4500087).

### Immunofluorescence

Hela VCPIP1 knockout cell lines were cultured and seeded in the chamber slides. When cells reached 70% confluency, the cells were fixed with 4% paraformaldehyde for 15 min at room temperature. Cells on slides were permeabilized with PBST containing 0.1% Triton X‐100 and 5% BSA for 30 min. The fixed cells were incubated with the Golgi apparatus's first primary antibody (GM130, 1:3000 dilution, CST,12 480) diluted in PBS containing 3% BSA at 4 °C overnight. After washing with PBS, cells were incubated for 1 h at room temperature in the dark with the secondary antibody conjugated to IgGcy3 (Jackson ImmunoResearch, 611‐165‐215) diluted in PBS and 3% BSA. The cells were washed with PBS again and stained with DAPI (1 µg mL^−1^) in PBS for 2 min at room temperature. After the final wash with PBS, the cells were mounted using a microslide, and the slides were sealed with nail polish. The cell images were captured and analyzed using the TCS SP8 confocal microscope equipped with LAS X software (Leica).

### Co‐Immunoprecipitation (Co‐IP)

For Co‐IP, the Flag‐tagged VCPIP1 and Myc‐tagged P97/VCP plasmids were transfected into HEK293T cells using Lipofectamine 2000 (Thermo Fisher Scientific, 11 668 019), after 48 h transfection, the cells were lysed in the ice‐cold cell lysis buffer (PBS with 1% protease inhibitor cocktail and 0.001% Triton X‐100), and disrupted by ultrasound. After centrifuging at 12 000 rpm for 15 min, the supernatant was then incubated with Anti‐Flag Magnetic Agarose beads (Thermo Fisher Scientific, A36797) for 4 h at 4 °C. The beads were washed with the cell lysis buffer and re‐suspended with SDS‐PAGE sample buffer. The prepared samples were separated by 8% SDS‐PAGE and analyzed by western blot using anti‐Flag (1:1000 dilution, Sigma, F1804) and anti‐Myc tag antibodies (1:1000 dilution, CST, PA1‐981).

## Conflict of Interest

The authors declare no conflict of interest.

## Author Contributions

T.L., R.L., P.L., Y.L., and R.Y. contributed equally to this work. F.L. and H.G. conceived and supervised the project. T.L., R.L., R.Y., Z.W., R.W., and H.G. performed plasmids construction, protein purification, and biochemical analysis. R.L. and Z.H. performed all the cell biology assays. P.L., Y.L., and H.G. performed Cryo‐EM grids preparation, data collection, and structure data analysis. L.Y. and Z.H. participated in biochemical assay design and manuscript preparation. F.L. and H.G. built the structural models and wrote the manuscript.

## Supporting information

Supporting Information

## Data Availability

The data that support the findings of this study are available in the supplementary material of this article.
