# Supplementary material for: Molecular Basis of VCPIP1 and P97/VCP Interaction Reveals Its Functions in Post‐Mitotic Golgi Reassembly
Source: Adv Sci (Weinh). 2024 Sep 5;11(41):2403417. doi: 10.1002/advs.202403417 (PMC11538695; doi:10.1002/advs.202403417)
Supplement: Supplementary file 1 — Supporting Information [file ADVS-11-2403417-s001.docx]

**Supporting Information for**

**Molecular Basis of VCPIP1 and P97/VCP Interaction Reveals Its Functions In Post-mitotic Golgi Reassembly**

*Tianzhui Liao^1#^, Ruotong Li^1#^, Ping Lu^2,3#^, Yusong Liu^2,3#^, Rong Yang^4#^, Hao Guo^1^, Zhuoxi Wu^1^, Ruiwen Wang^1^, Ling Yuan^1^, Zhengmao Hu^1^, Haishan Gao^2,3*^, Faxiang Li^1*^*

1 MOE Key Laboratory of Rare Pediatric Diseases, Center for Medical Genetics, School of Life Sciences, Central South University, Changsha, China.

2 Zhejiang Key Laboratory of Structural Biology, Westlake University, Hangzhou, Zhejiang, China.

3 Westlake Laboratory of Life Sciences and Biomedicine, Hangzhou, Zhejiang, China.

4 State Key Laboratory of Developmental Biology of Freshwater Fish, Engineering Research Center of Polyploid Fish Reproduction and Breeding of the State Education Ministry, College of Life Sciences, Hunan Normal University, Changsha, China.

# These authors contributed equally to this work

*Correspondence: chinalfx@163.com (F.L.), gaohaishan@westlake.edu.cn (H.G.).

**Supplementary figures and tables**


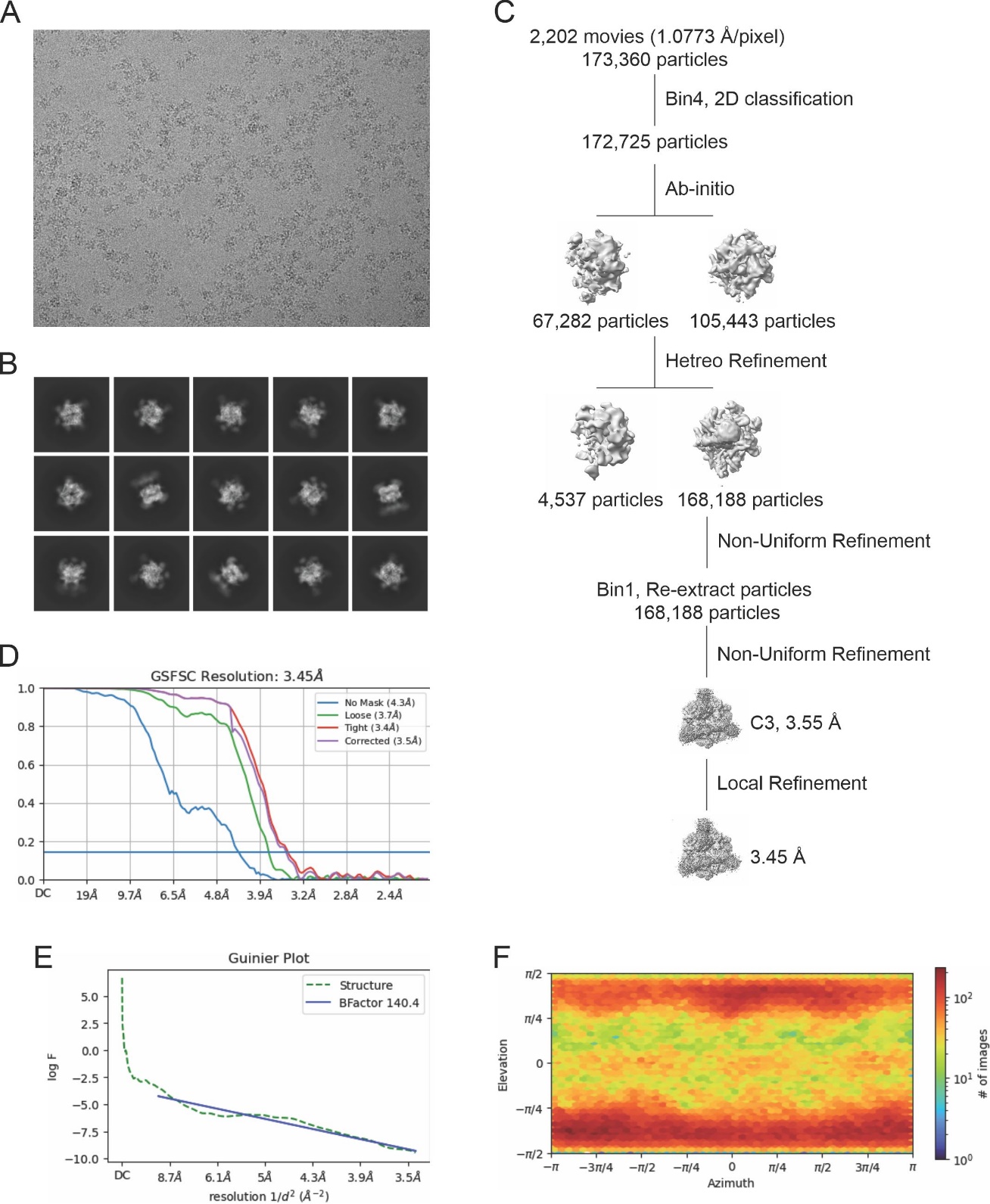


**Fig. S1. Cryo-EM analysis of P97/VCP-VCPIP1 complex.** (A) Representative raw cryo-EM micrograph of the P97/VCP-VCPIP1 complex after motion correction. (B) Representative 2D class averages. (C) Workflow of cryo-EM data processing to obtain the 3D reconstructions of P97/VCP-VCPIP1 complex. (D) The Gold-standard FSC curve of the final EM map for the P97/VCP-VCPIP1 complex. The resolution for this reconstruction was determined using FSC=0.143 criterion. (E) The Guinier Plot for the final reconstruction of the final EM map for VCP-VCPIP1 complex. (F) The angular distribution of particles used in the final reconstruction of the final EM map for VCP-VCPIP1 complex.


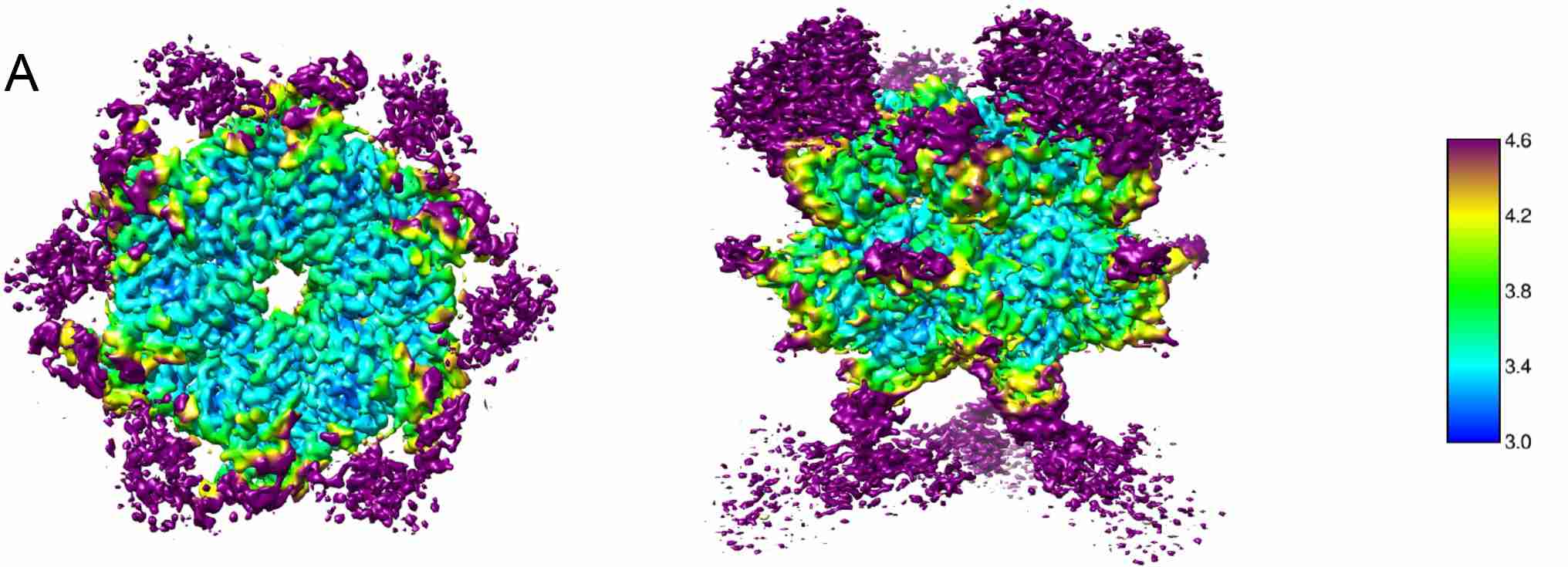


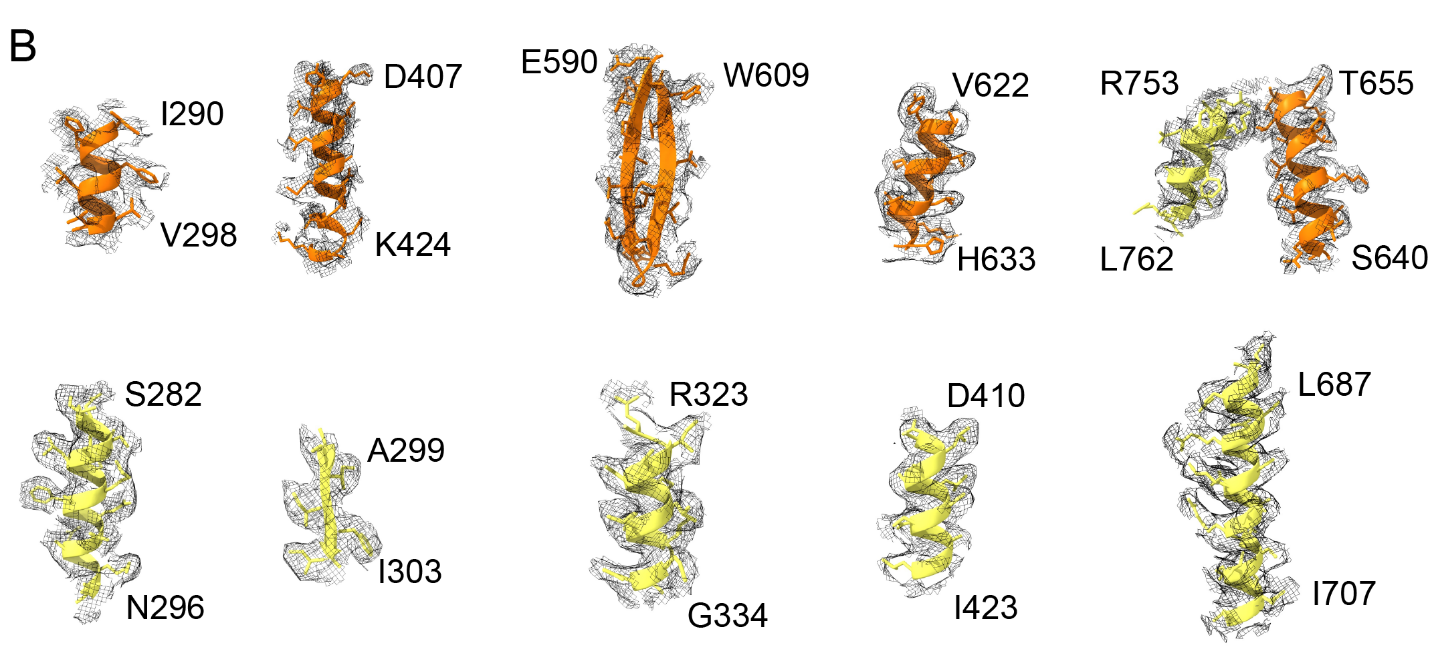


**Fig. S2. Characterization of the Cryo-EM map and models for P97/VCP-VCPIP1 complex.** (A) Local resolution maps of P97/VCP-VCPIP1 complex in top (left) and side (right) view. (B) The representative cryo-EM density maps of key structural elements for human VCP (yellow) and VCPIP1 (orange).


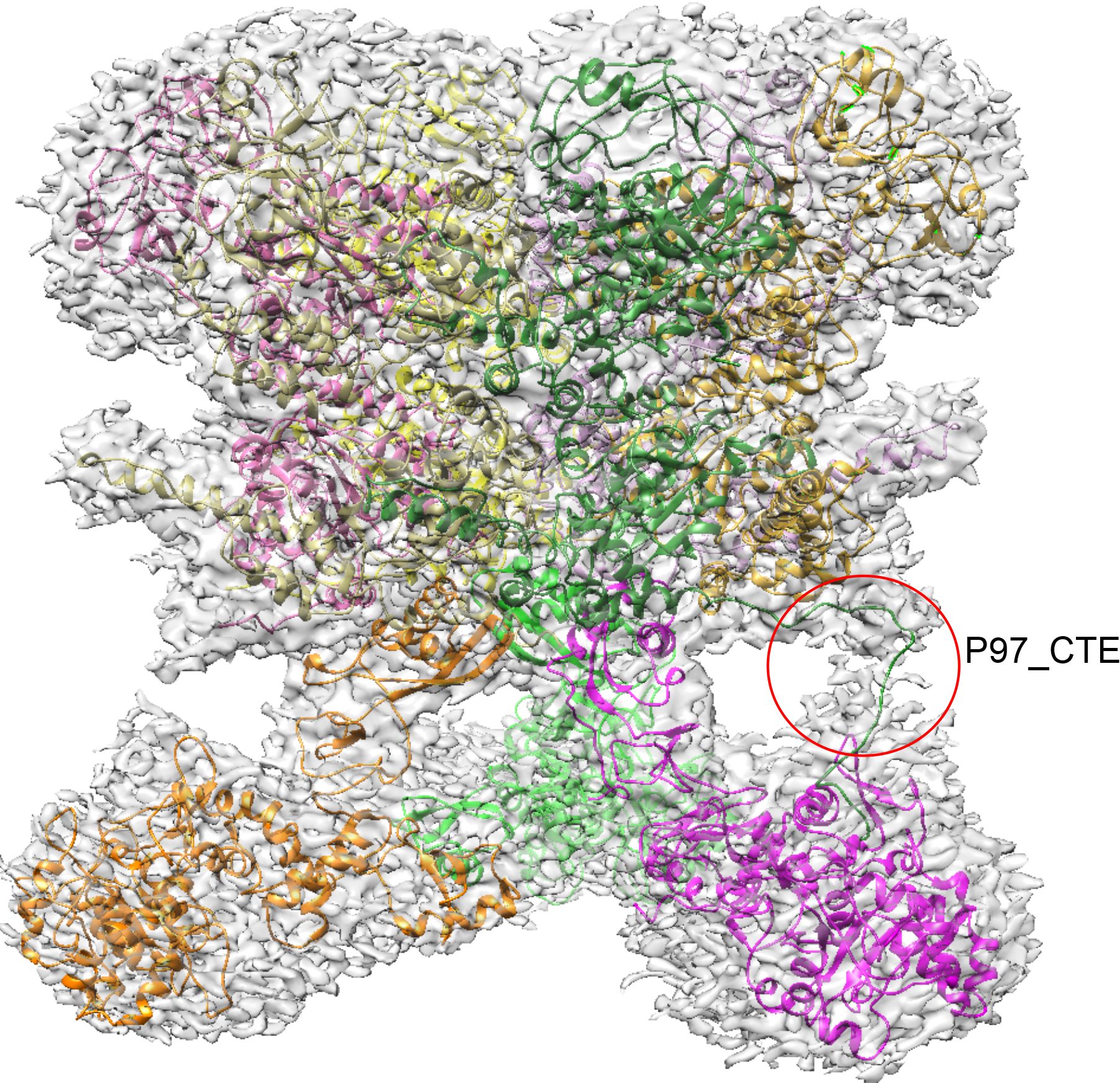


**Fig. S3. The electron density map of P97/VCP-VCPIP1 complex before imposing C3 Symmetry. An unfitted, continuous weak density linking P97/VCP to VCPIP1 that belonged to the C-termianl extension region of P97/VCP was indicated by the red cycle.**


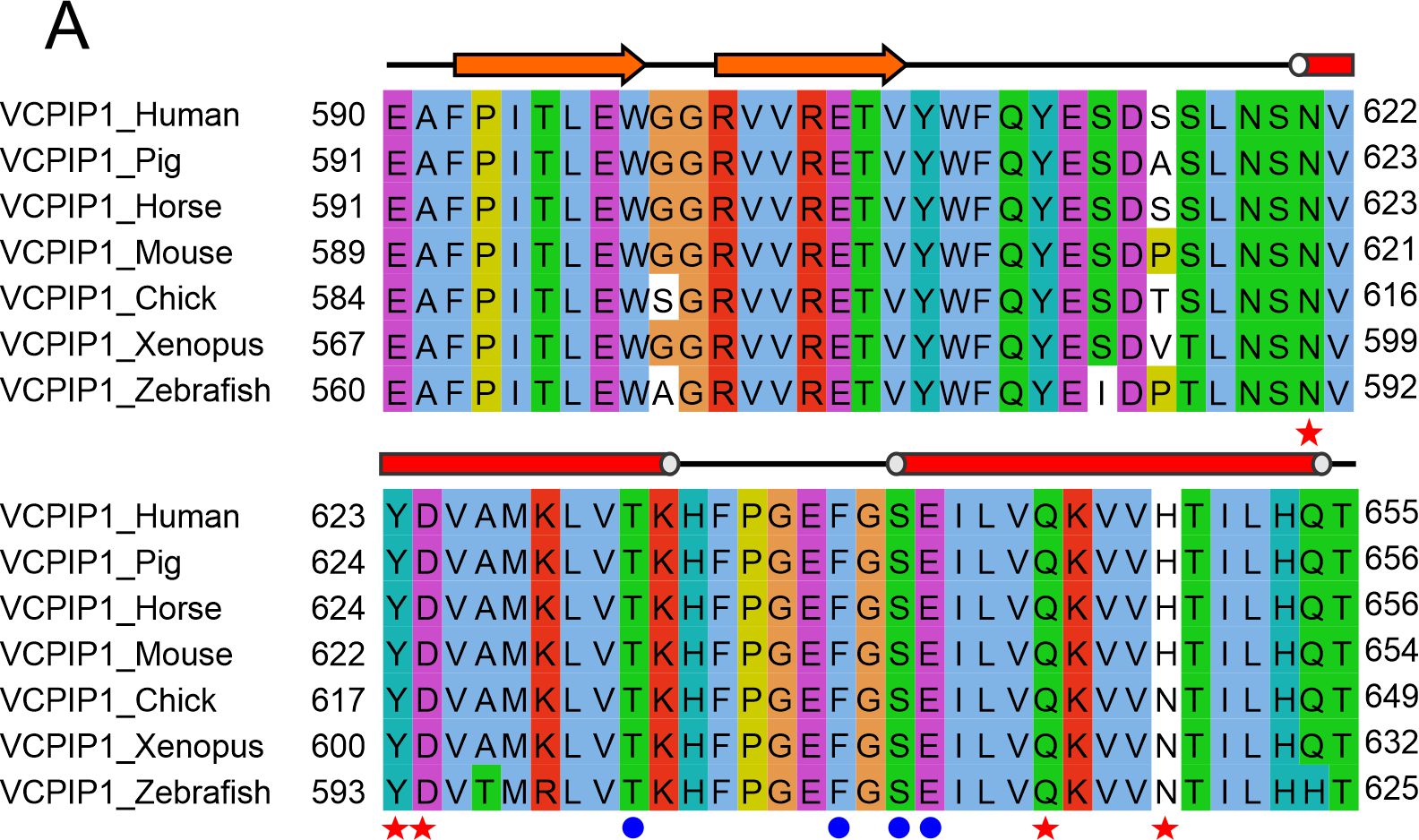


**Fig. S4. The conservation analysis of VCPIP1 UFD1 domain.** (A) Sequence alignment of the UFD1 domain of VCPIP1 from different species, with the secondary structure elements of UFD1 shown at the top. The conserved residues are highlighted with the software Jalview2.8.1 (http://www.jalview.org/). Key residues required for the two adjacent P97/VCP protomers binding are indicated by the red stars and blue dots.


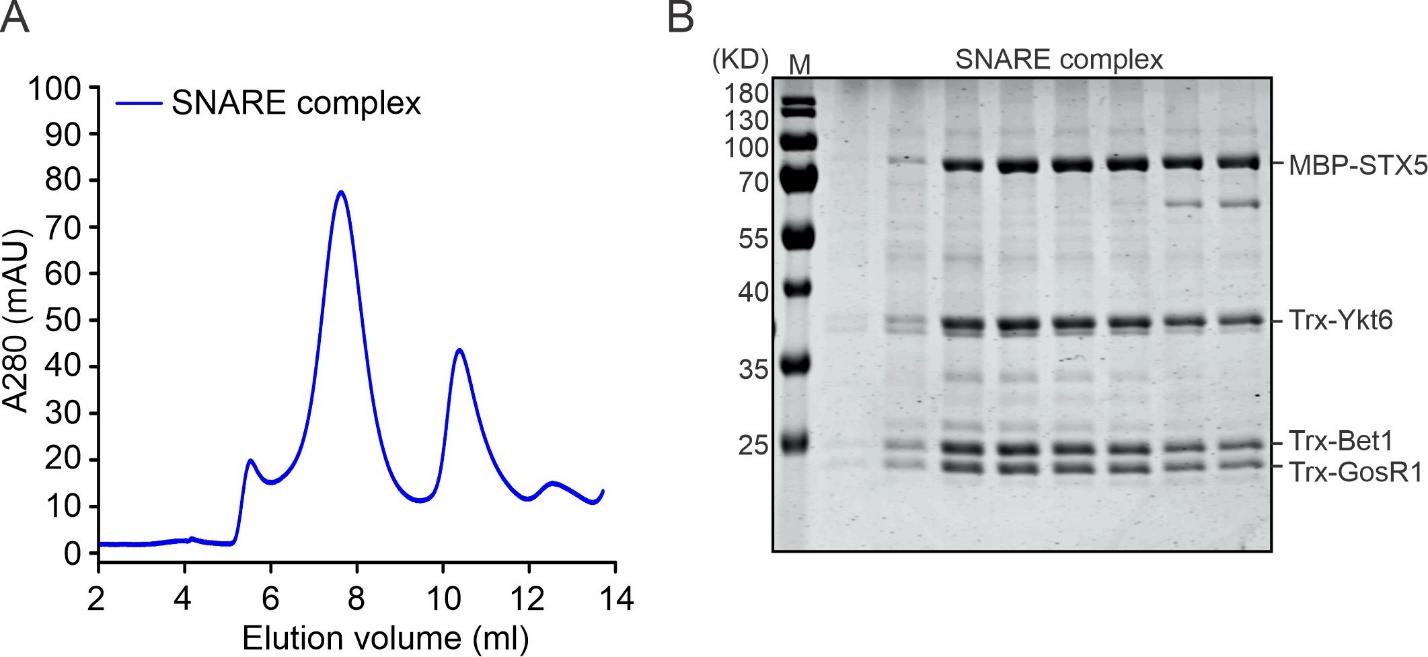


**Fig. S5. Purification of Syntaxin5-containing SNARE complex.** (A) Gel filtration profile of Syntaxin5-containing SNARE complex. (B) The SDS-PAGE combined Coomassie Brilliant Blue staining showing the SNARE complex fractions in panel (A).


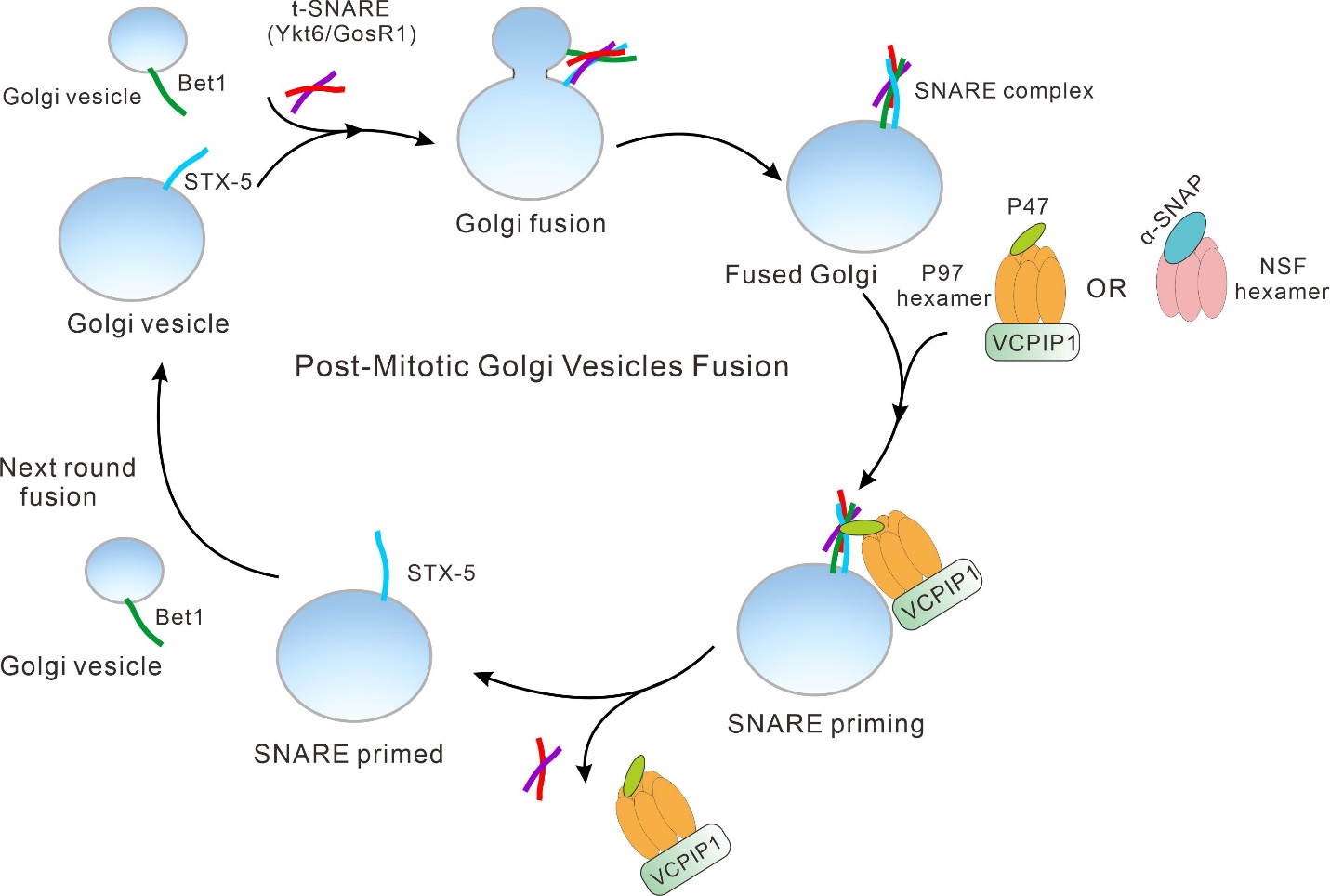


**Fig. S6. A proposed cartoon model illustrating the process of post-mitotic Golgi fusion mediating by the VCPIP1-P97/VCP-P47 complex.**

**Table S1. Cryo-EM data collection, refinement and validation statistics**

|  | VCP-VCPIP1  (EMDB-39360) (PDB 8YKA) |
| --- | --- |
| **Data collection and processing** |  |
| Magnification | 81,000× |
| Voltage (kV) | 300 |
| Electron exposure (e–/Å^2^) | 50 |
| Defocus range (μm) | -1 – 2.0 |
| Pixel size (Å) | 1.0773 |
| Symmetry imposed | C3 |
| Final particle images (no.) | 168,188 |
| Map resolution (Å)  FSC threshold | 3.45  0.143 |
|  |  |
| **Refinement** |  |
| Model resolution (Å)  FSC threshold | 3.8  0.5 |
| Map sharpening *B* factor (Å^2^) | 140.4 |
| Model composition  Non-hydrogen atoms  Protein residues  Ligands | 48386  6291  0 |
| *B* factors (Å^2^)  Protein | 77.61 |
| R.m.s. deviations  Bond lengths (Å)  Bond angles (°) | 0.006  0.813 |
| Validation  MolProbity score  Clashscore  Poor rotamers (%) | 2.22  16.04  0.24 |
| Ramachandran plot  Favored (%)  Allowed (%)  Disallowed (%) | 91.30  8.64  0.06 |
